# The Effects of FIN56 and Free Iron Species on Ferroptosis and Mitochondrial Activity in Adult Human Astrocytes

**DOI:** 10.1002/alz70855_104099

**Published:** 2025-12-24

**Authors:** Morgan Stetzer, Bethany Bass, Andrea C. Jimenez‐Vergara, Luke Chandler, German Plascencia‐Villa, George Perry, Dany J. Munoz‐Pinto

**Affiliations:** ^1^ Trinity University, San Antonio, TX, USA; ^2^ The University of Texas at San Antonio (UTSA), San Antonio, TX, USA; ^3^ The University of Texas at San Antonio, San Antonio, TX, USA

## Abstract

**Background:**

Ferroptosis is a type of cell death induced by iron dysregulation. It is characterized by mitochondrial abnormalities such as smaller size, outer membrane rupture, dense membranes, reduction in mitochondrial crista, and reduced mitochondrial membrane potential. Recent literature has identified ferroptosis as a mechanism contributing to neuronal death and the progression of neurological disorders such as Alzheimer's disease (AD). Ferroptosis has been primarily studied on neurons or neuron‐like cells, but limited research has been conducted using astrocytes. Astrocytes are glial cells recognized, among other functions, for maintaining the blood‐brain barrier, regulating synapses, repairing tissue, and recently have been acknowledged as significant players in the onset and progression of neurological disorders including AD.

**Methods:**

In this work, we evaluated the effects of N2, N7‐dicyclohexyl‐9‐(hydroxyimino)‐9H‐fluorene‐2,7‐disulfonamide (FIN56) and two free iron sources (FeCl_2_ and FeCl_3_) as ferroptosis inducing agents on the biological behavior of adult human astrocytes. The response of the cells to FIN56 and free iron species at different concentrations, and exposure times was evaluated for cell viability, cell morphology, reactive oxygen species (ROS) production, and mitochondrial function.

**Results:**

Initial results showed that human astrocyte viability was not significantly affected by the presence of free iron up to 50 μM. In addition, JC‐1 measurements revealed that mitochondrial activity was enhanced in human astrocytes cultured in free iron supplemented medium. On the other hand, the presence of FIN56 in the culture medium appeared fatal to cells event at low concentrations. This cell death was linked to significantly reduced mitochondrial membrane potential caused by FIN56.

**Conclusion:**

At the same molar concentration, we observed a stronger and detrimental impact on astrocyte survival and mitochondrial function with the use of FIN56 relative to free iron species.

